# Sirtuins at the Crossroads between Mitochondrial Quality Control and Neurodegenerative Diseases: Structure, Regulation, Modifications, and Modulators

**DOI:** 10.14336/AD.2022.1123

**Published:** 2023-06-01

**Authors:** Hui Xu, Yi-Yang Liu, Lin-Seng Li, You-Shuo Liu

**Affiliations:** ^1^Department of Geriatrics, The Second Xiangya Hospital of Central South University, Changsha, Hunan, China.; ^2^Institute of Aging and Age-related Disease Research, Central South University, Changsha, Hunan, China.

**Keywords:** Sirtuins, structure, regulation, mitochondrial quality control, neurodegenerative disease, sirtuin modulators

## Abstract

Sirtuins (SIRT1-SIRT7), a family of nicotinamide adenine dinucleotide (NAD^+^)-dependent enzymes, are key regulators of life span and metabolism. In addition to acting as deacetylates, some sirtuins have the properties of deacylase, decrotonylase, adenosine diphosphate (ADP)-ribosyltransferase, lipoamidase, desuccinylase, demalonylase, deglutarylase, and demyristolyase. Mitochondrial dysfunction occurs early on and acts causally in the pathogenesis of neurodegenerative diseases, including Alzheimer’s disease (AD), Parkinson’s disease (PD), and Huntington’s disease (HD). Sirtuins are implicated in the regulation of mitochondrial quality control, which is highly associated with the pathogenesis of neurodegenerative diseases. There is growing evidence indicating that sirtuins are promising and well-documented molecular targets for the treatment of mitochondrial dysfunction and neurodegenerative disorders by regulating mitochondrial quality control, including mitochondrial biogenesis, mitophagy, mitochondrial fission/fusion dynamics, and mitochondrial unfolded protein responses (mtUPR). Therefore, elucidation of the molecular etiology of sirtuin-mediated mitochondrial quality control points to new prospects for the treatment of neurodegenerative diseases. However, the mechanisms underlying sirtuin-mediated mitochondrial quality control remain obscure. In this review, we update and summarize the current understanding of the structure, function, and regulation of sirtuins with an emphasis on the cumulative and putative effects of sirtuins on mitochondrial biology and neurodegenerative diseases, particularly their roles in mitochondrial quality control. In addition, we outline the potential therapeutic applications for neurodegenerative diseases of targeting sirtuin-mediated mitochondrial quality control through exercise training, calorie restriction, and sirtuin modulators in neurodegenerative diseases.

## 1. Introduction

Neurodegenerative diseases, a heterogeneous class of diseases, are characterized by a slow, progressive loss of selective neuronal cell populations and neuronal dysfunction [1]. Aging is a primary risk factor for neurodegenerative diseases, including Alzheimer’s disease (AD), Parkinson’s disease (PD), and Huntington’s disease (HD). As the population ages, the incidence and prevalence of neurodegenerative diseases continue to rise. Global statistics suggest that AD is increasing steadily with age; it is estimated to affect up to 5.8 million Americans and more than 50 million individuals worldwide [2]. The prevalence of neurodegenerative diseases has grown enormously and poses an increasing financial burden and challenge to society, families, and individual patients [3]. Globally, PD is the second most common neurodegenerative disease (after AD), affecting more than 6 million individuals [4]. Approximately 10.6 to 13.7 people per 100,000 in Western populations suffer from HD [5]. Extensive studies have explored the pathogenesis of and interventions for neurodegenerative disorders. There are, however, not fully effective or definitive pharmacological treatments for these diseases.

Mitochondria are major contributors to energy metabolism, apoptosis, and cellular signaling. As highly metabolically active cells, neurons are particularly sensitive to alterations in mitochondrial function due to the high amount of energy required for neuronal activity [6]. It is remarkable that in all major examples of these diseases, mitochondrial dysfunction occurs early on and acts causally in the pathogenesis of the disease [7, 8]. Mitochondria isolated from the animal and postmortem human brain tissues exhibit increased functional heterogeneity with age, accompanied by increased oxidative damage, decreased electron transport chain function, disruption of membrane potential, impaired Ca^2+^ homeostasis, and/or the accumulation of dysfunctional mitochondria, all of which may contribute to the pathogenesis of neurodegenerative diseases [8]. Robust mitochondrial function is more critical for neurons than other cells. Mitochondrial quality control is key to maintaining the integrity and function of mitochondria [9]. Therefore, unraveling the molecular etiology of dysregulated mitochondrial quality control may provide new prospects for the treatment and prevention of neurodegenerative diseases.

**Figure 1.**
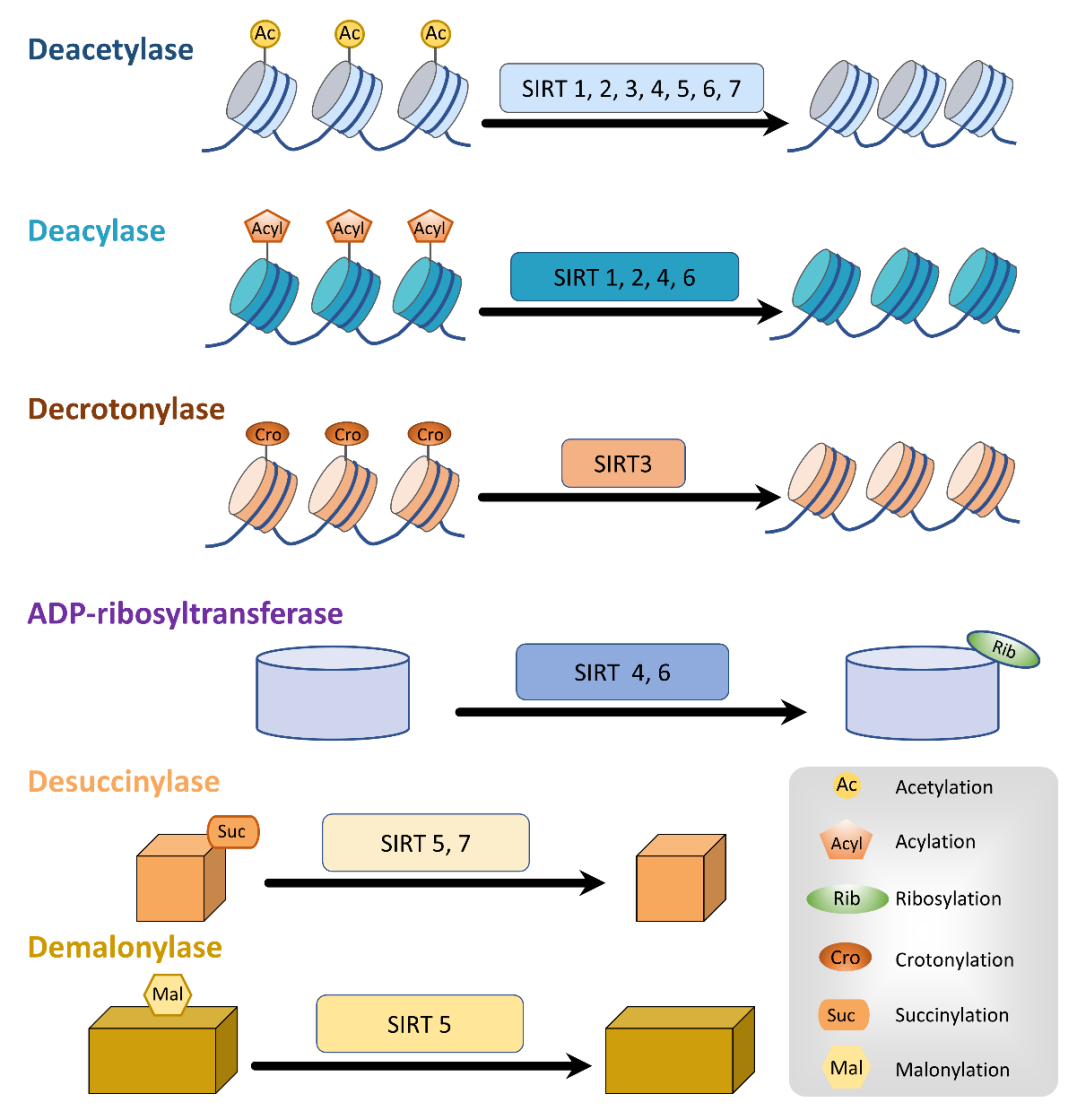
Multiple enzymatic functions of sirtuins. Sirtuins are a family of NAD^+^-dependent enzymes. SIRT1, SIRT2, SIRT4, and SIRT6 display deacylase activity. SIRT3 exhibits decrotonylase activity. SIRT4 and SIRT6 exhibit ADP-ribosyl transferase activity. SIRT5 is a cofactor in the desuccinylation and demalonylation of target proteins. SIRT7 show desuccinylase activity.

Mammalian sirtuins are a class of highly conserved nicotinamide adenine dinucleotide (NAD^+^)-dependent enzymes [10]. Although their primary function is deacetylation, some sirtuins also have decrotonylase, adenosine diphosphate (ADP)-ribosyltransferase, lipoamidase, desuccinylase, demalonylase, deglutarylase, and demyristolyase [11] (Fig. 1). Mass spectrometry studies hint that a variety of mitochondrial proteins implicated in metabolism and energy production are acetylated, which suggests that sirtuins potentially regulate multiple mitochondrial biological processes [12]. Sirtuins have aroused widespread interest as crucial regulators of mitochondrial quality control and neurodegeneration, such as the changes seen in AD, PD, and HD [13]. Nevertheless, the underlying role of sirtuin-mediated mitochondrial quality control in the pathogenesis of neurodegenerative disorders is still not well elucidated. Therefore, the fundamental purpose of this review is to outline the current understanding of sirtuins and shed light on how various aspects of sirtuin-mediated mitochondrial quality control, including mitochondrial biogenesis, mitophagy, mitochondrial dynamics, and mitochondrial unfolded protein response (mtUPR), regulate the pathogenesis of neurodegenerative disease. In addition, we establish the efficacy of sirtuins as molecular therapeutic targets for restoring mitochondrial quality control in neurodegenerative diseases.

## 2. What is it that causes the divergent biological functions of each isoform of the sirtuin family?

Sirtuins are the mammalian homologs of yeast silent information regulator 2 (Sir2), a key modulator of longevity and cell senescence in *Saccharomyces cerevisiae* [14]. To date, seven members of the sirtuin family have been identified: SIRT1-SIRT7. There was an overlap in enzymatic activity among different sirtuin isoforms. Besides, some sirtuins can act on the same targets, such as p53. Thus, what accounts for the divergent biological functions of each isoform in this family?

**Figure 2.**
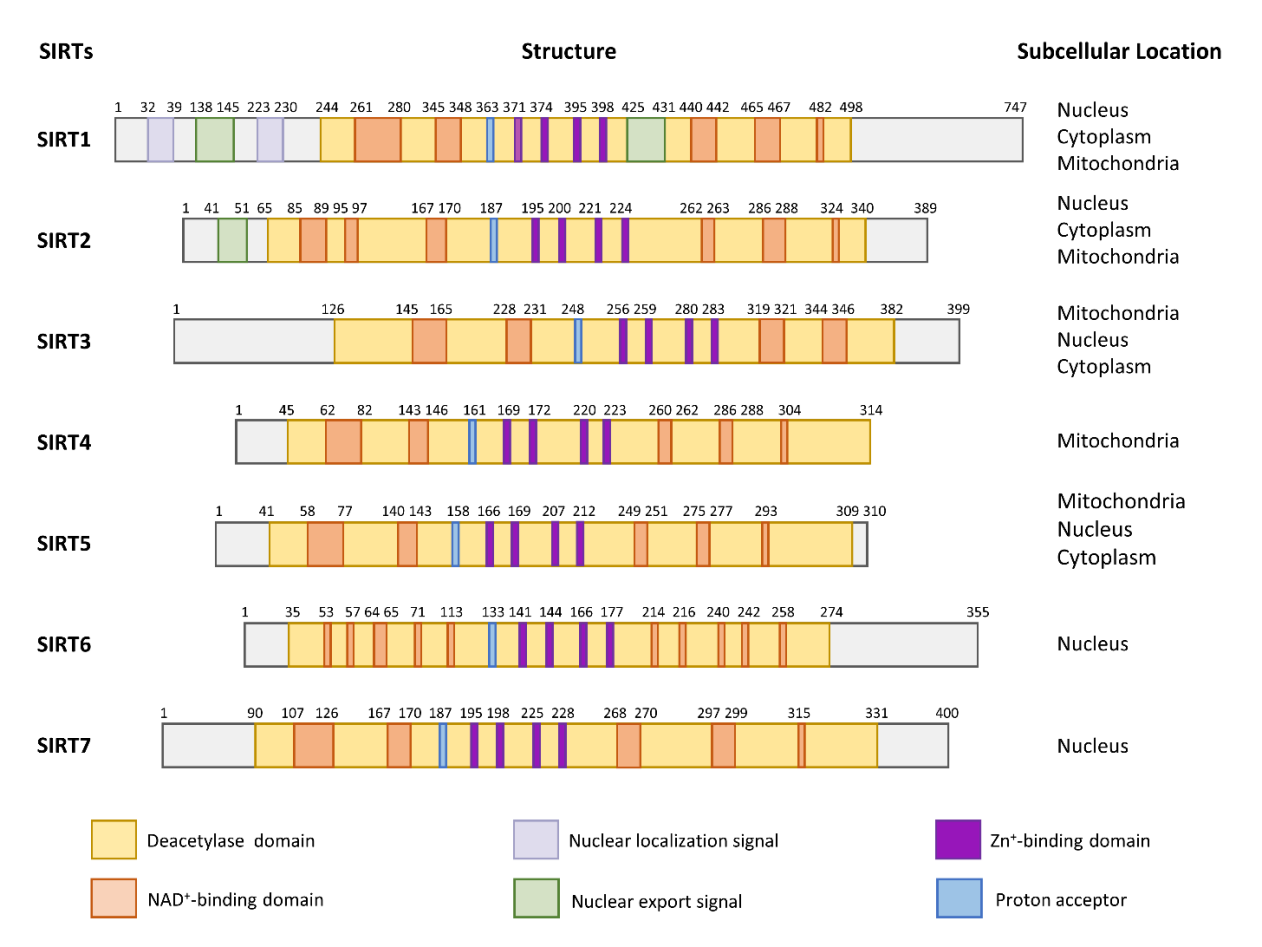
Structure and subcellular localization of human sirtuins. The positions of the amino acid are indicated on each schematic. Domains are shown in different colors.

### 2.1 Structure, subcellular localization, and enzymatic activity

Sirtuins share a conserved catalytic core domain that comprises a highly conserved NAD^+^-binding domain that is primarily folded by Rossmann and a less conserved domain containing zinc finger structures (Fig. 2). The cracks between the NAD^+^-binding and structural zinc-binding domains provide substrate-binding sites. Although these members are relatively conserved, they contain diverse N- and C-terminal extensions [15]. Given their different expression and subcellular localization, unique binding substrates, and distinct enzymatic activities, each member of the sirtuin family exhibits varied physiological activities.

#### 2.1.1 SIRT1

The human *SIRT1* gene is located on chromosome 10q21.3, contains eight introns and nine exons, and encodes a protein that comprises 747 amino acids. SIRT1 is predominantly localized in the nucleus and has a broad range of roles in the regulation of multiple fundamental processes associated with aging, including metabolism, inflammation, stress, apoptosis, and DNA repair [16].

#### 2.1.2 SIRT2

The *SIRT2* gene is located on chromosome 19q13.2 and consists of 21,064 bases. The total length of SIRT2 contains 389 amino acids. It comprises a central catalytic domain of 232 amino acids that is flanked by N- and C-terminal helical extensions [17]. SIRT2 exists in two forms: a protein with a molecular weight of about 43 kDa and a short form with a molecular weight of about 39.5 kDa. SIRT2 is mainly located in the cytoplasm, but it is also present in the nucleus and mitochondria [18]. It is the most abundant member of the sirtuin family in the aging central nervous system [19]. In addition to deacetylation, SIRT2 also has defatty-acylase and deacylase activities [17].

#### 2.1.3 SIRT3

SIRT3 is encoded by the nuclear genome locus on chromosome 11p15.5 and is about 21 kb in length. It is composed of a catalytic core of approximately 275 amino acid residues flanked by N- and C-terminal extensions. Two isomers of the SIRT3 protein are encoded: a full-length protein of about 44 kDa and a short form of approximately 28 kDa [20]. Macromolecule SIRT3 is localized in the mitochondria, nucleus, and cytoplasm, whereas the short-form SIRT3 exists exclusively in mitochondria [21]. SIRT3 regulates a wide spectrum of mitochondrial functions by catalyzing the deacetylation of mitochondrial proteins.

#### 2.1.4 SIRT4

The human *SIRT4* gene is located on q24.31 of chromosome 12 and encodes a protein of about 35.2 kDa. Sequence and structural analysis of SIRT4 indicated that it contains the requisite amino acids involved in the deacylase reactions. SIRT4 is composed of a homologous sirtuin deacylase domain, a conserved catalytic histidine (H161), and a NAD^+^-binding region [22]. It is not only present in the mitochondria but also in the nucleus and cytoplasm [23]. SIRT4 has specific deacetylase [24], lipoamidase [25], ADP-ribosyltransferase [26], and deacylase activities [22].

#### 2.1.5 SIRT5

SIRT5 is located on chromosome 6p23. The *SIRT5* gene encodes four SIRT5 protein isomers, of which SIRT5^iso1^ and SIRT5^iso2^ are the most studied. SIRT5^iso1^ contains 310 amino acid residues, whereas SIRT5^iso2^ comprises 299 amino acids. SIRT5 consists of a large domain and a small domain. The large domain is a NAD^+^-binding motif folded by Rossmann and the small domain is also called the zinc-binding domain [27]. SIRT5 is a mitochondrial protein with a variety of enzymatic functions, including deacetylation [28], desuccinylation [29], demalonylation [30], and deglutarylation [31].

#### 2.1.6 SIRT6

*SIRT6* is located at p13 of chromosome 19 (19p13.3). It has a molecular weight of about 39 kDa and encodes a protein composed of 355 amino acid residues. Two isomers of SIRT6 exist, encoded by seven and eight exons, respectively. SIRT6 lacks the NAD^+^-binding region and only contains one divergent zinc-binding domain, which is slightly different from other sirtuins [32]. Note that SIRT6 has a stable helical structure for NAD^+^ binding. It is found in the nucleus and has multiple enzymatic activities, including deacetylase [33], mono-ADP-ribosyltransferase [34], and defatty-acylase [35].

#### 2.1.7 SIRT7

The human *SIRT7* gene is located on chromosome 17q25.3 and comprises 9,385 bases. The protein encoded by the *SIRT7* gene contains 400 amino acids with a molecular mass of about 44.9 kDa. Like other sirtuins, SIRT7 retains the central catalytic domain, whereas the flanking N- and C-terminal extensions are unique. SIRT7 is abundant in the nucleus and is highly enriched in the nucleolar compartment [36]. There are also small amounts of SIRT7 in the cytoplasm. SIRT7 has the properties of deacetylase [37], desuccinylase [38], mono-ADP-ribosyltransferase [39], defatty-acylase [40], decrotonylase [41], and deglutarylase [42].

### 2.2 Transcriptional regulation of sirtuins

**Figure 3.**
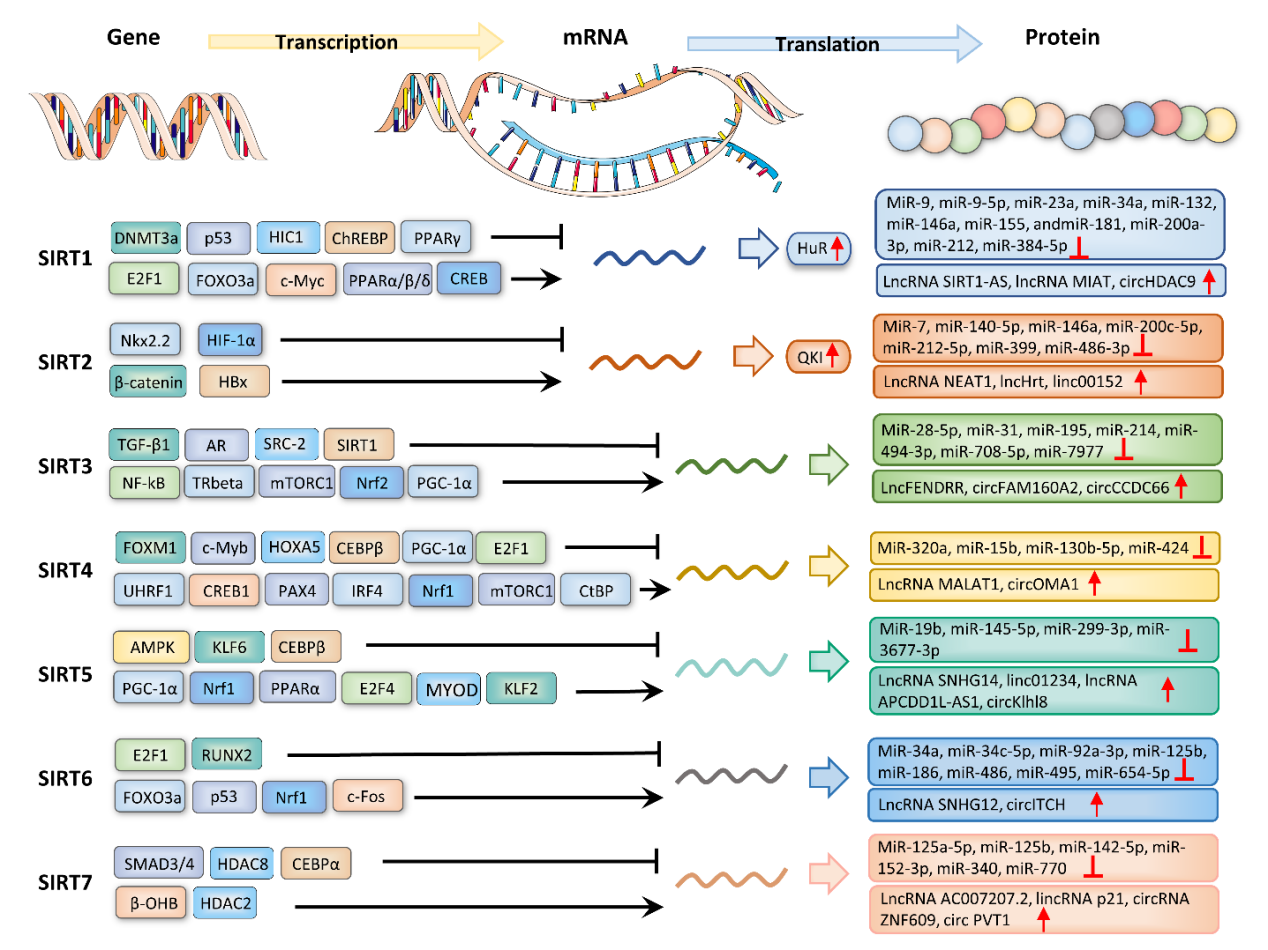
Mechanisms of action that regulate sirtuin expression. The regulatory mechanisms of sirtuin expression include transcriptional regulation and post-transcriptional regulation.

Transcriptional regulation in eukaryotes occurs in the proximal region of the promoter. Sirtuins play a significant role in regulating cell metabolism and cellular functions such as inflammatory responses, oxidative stress, cell senescence, and apoptosis. Nevertheless, how they are transcriptionally regulated is still not well elucidated (Fig. 3).

#### 2.2.1 SIRT1

The transcription of SIRT1 is regulated by DNA methylation and several transcription factors. Under certain pathological conditions, DNA hypermethylation is associated with decreased SIRT1 expression, which can be enhanced through treatment with DNA methylation inhibitors [43]. Moreover, SIRT1 transcription is modulated by multiple negative feedback loops. The transcription factors p53 [44] and hypermethylated in cancer 1 (HIC1) [45] suppressed SIRT1 transcription, whereas E2F transcription factor-1 (E2F1) [46], forkhead box class O3a (FOXO3a), and c-Myc [47] promoted SIRT1 transcription. In turn, SIRT1 directly deacetylated p53, HIC1, E2F1, FOXO3a, and c-Myc. Additionally, peroxisome proliferator-activated receptor α (PPARα) [48], PPARβ/δ [49], and cAMP response element-binding (CREB) [50] upregulated SIRT1 transcription, whereas carbohydrate response element-binding protein (ChREBP) [50] and PPARγ [51] decreased SIRT1 levels.

#### 2.2.2 SIRT2

SIRT2 transcriptional regulation can occur under different physiological and pathological conditions. Inoue et al. reported that histone deacetylation of the 5′ untranslated region (UTR) of SIRT2 was involved in the downregulation of SIRT2 in glioma cells [52]. Besides, the transcription factor NK2 homeobox 2.2 (Nkx2.2) and hypoxia-inducible factor 1α (HIF-1α) negatively regulated SIRT2 transcription. Nkx2.2 inhibited SIRT2 expression by interacting with the SIRT2 promoter via histone deacetylase 1 (HDAC-1) [53], whereas HIF-1α bound to the hypoxia response element in the SIRT2 promoter [54]. Li et al. discovered that suppression of the Wnt/β-catenin pathway elevated the promoter activity and mRNA expression of SIRT2. SIRT2 expression is regulated transcriptionally by β-catenin through interacting with its promoter [55]. Moreover, the hepatitis B viral protein HBx participated in the enhancement of the mRNA and protein levels of SIRT2 by binding to the SIRT2 promoter [56].

#### 2.2.3 SIRT3

The transcription factor nuclear factor kappa B (NF-kB) [57], thyroid hormone receptor beta (TRbeta) [58], mechanistic target of rapamycin complex 1 (mTORC1) signal [59], and nuclear respiratory factor 2 (Nrf2) [60] promoted SIRT3 transcription by enhancing activation of the SIRT3 promoter. A surprising finding by Kanwal et al. showed that mitochondrial SIRT3 and nuclear SIRT6 regulated each other’s activity [61]. SIRT6 regulates SIRT3 levels by strengthening Nrf2-dependent SIRT3 gene transcription. Mechanistically, on the one hand, SIRT6 interacted with Nrf2 and antagonized its interaction with Kelch-like ECH-associated protein 1 (Keap1), a negative regulator of Nrf2. On the other hand, SIRT6 alleviated Keap1 expression. Conversely, androgen receptor (AR) and its coregulator steroid receptor coactivator-2 (SRC-2) inhibited SIRT3 transcription by recruiting histone deacetylase 2 to the SIRT3 promoter [62]. Proliferator-activated receptor γ coactivator 1α (PGC-1α) promoted SIRT3 gene transcription through activating estrogen-related receptor-α (ERR-α) and binding to the proximal promoter region of SIRT3 [63]. Moreover, Carnevale et al. reported that SIRT1 deacetylated the ZF5 region on the SIRT3 promoter, leading to SIRT3 transcription inhibition [64]. It is intriguing that a lack of CR6-interacting factor 1 (CRIF1) triggered ubiquitination-mediated degradation of PGC-1α and Nrf2, eventually reducing SIRT3 expression [65]. Furthermore, transforming growth factor-β1 (TGF-β1) repressed SIRT3 transcription and decreased its deacetylase activity in lung fibroblasts [66].

#### 2.2.4 SIRT4

Transcription factors, such as c-Myb [67], FOXM1 [68], homeobox A5 (HOXA5), E2F1, CCAAT/enhancer-binding protein β (CEBPβ), and PGC-1α [69], transcriptionally activated SIRT4 expression, whereas Nrf1, ubiquitin-like with plant homeodomain and ring finger domains 1 (UHRF1) [70], CREB1, paired box 4 (PAX4), interferon regulatory factor 4 (IRF4) [71], mTORC1 [72], and C-terminal-binding protein (CtBP) [73] transcriptionally suppressed SIRT4 expression. Besides, transcriptional activation of SIRT4 can be mediated by the demethylation of the SIRT4 promoter [67].

#### 2.2.5 SIRT5

At the transcriptional level, SIRT5 is under the control of AMP-activated kinase (AMPK) and PGC-1α, which have opposite effects on its mRNA levels. In one study, AMPK overexpression downregulated SIRT5 mRNA levels in mouse primary hepatocytes by 58%, whereas elevated PGC-1α upregulated SIRT5 mRNA and protein expression in an ERRα- and PPARα-dependent manner [74]. Moreover, during bovine adipocyte differentiation, SIRT5 transcription was negatively regulated by Kruppel-like factor 6 (KLF6) and CEBPβ and positively modulated by demethylation, E2F4, KLF2, Nrf1, MYOD, and PPARα [75, 76].

#### 2.2.6 SIRT6

The expression of SIRT6 can be regulated at the transcriptional level. Transcription factors E2F1 [77] and Runt-related transcription factor 2 (RUNX2) [78] were involved in inhibiting SIRT6 transcription. In contrast, FOXO3a [79, 80], c-Fos, and Nrf1 [81] promoted SIRT6 transcription and subsequently repressed glycolysis. In addition, the expression of SIRT6 mRNA was decreased in p53^-/-^ mice, indicating that p53 can interact with SIRT6 and activate its transcription [82].

#### 2.2.7 SIRT7

There are many challenges and opportunities in the field of SIRT7 transcriptional regulation. The SMAD3/4 complex and its co-factor HDAC8 negatively regulated SIRT7 transcription via local chromatin remodeling [83], whereas CEBPα inhibited SIRT7 transcription through recruiting HDAC3 [84]. Moreover, the activation of SIRT7 transcription was linked to alterations in acetylation, which can be influenced by the ketone body β-hydroxybutyrate (β-OHB)/HDAC2 signaling pathway [85].

### 2.3 Post-transcriptional regulation of sirtuins

A plethora of proteins is involved in regulating mRNA stability and translation [86]. Moreover, non-coding RNAs (ncRNAs), especially microRNAs (miRNAs), are involved in the post-transcriptional regulation of sirtuins. Mechanistically, miRNAs bind to the 3′ UTR of target mRNA to promote mRNA degradation or repress mRNA translation [87]. As competitive endogenous RNAs, long ncRNAs (lncRNAs) and circular RNAs (circRNAs) regulate the post-transcription of SIRT1 by sponging miRNAs [88].

#### 2.3.1 SIRT1

RNA binding protein human antigen R (HuR) was implicated in the stabilization of SIRT1 mRNA by binding to its 3′ UTR [86]. With cell senescence, reduced HuR expression leads to decreased SIRT1 expression levels. Alterations in miRNA patterns, such as miRNA-9, miRNA-9-5p, miRNA-23a, miRNA-34a, miRNA-132, miRNA-146a, miRNA-155, and miRNA-181, miRNA-200a-3p, miRNA-212, and miRNA-384-5p, seem to be responsible for the decreased expression of SIRT1 [89-97]. In addition, Li et al. pointed out that SIRT1 antisense lncRNA promoted SIRT1 stabilization and improved SIRT1 mRNA and protein levels through competing with miRNA-34a to bind to the SIRT1 3′ UTR [98, 99]. Besides, the post-transcription of SIRT1 was also regulated by the lncRNA MIAT/miRNA-132 and circHDAC9/miRNA-138 axes [100, 101].

#### 2.3.2 SIRT2

By directly binding to the quaking response element (QRE) located in the 3′ UTR of SIRT2 mRNA, the RNA-binding protein quaking (QKI) upregulated the expression and stability of SIRT2 mRNA [102]. Additionally, several miRNAs and lncRNAs were implicated in the post-transcriptional regulation of SIRT2. MiRNAs, such as miRNA-7, miRNA-140-5p, miRNA-146a, and miRNA-486-3p negatively regulated SIRT2 expression levels [103-106]. LncRNA NEAT1 positively modulated the mRNA stability and expression of SIRT2 by sponging miRNA-221-3p [107].

#### 2.3.3 SIRT3

It has been reported that many ncRNAs are associated with the post-transcription of SIRT3. Multiple miRNAs, such as miRNA-28-5p, miRNA-31, miRNA-195, miRNA-214, and miRNA-494-3p negatively regulated SIRT3 expression [108-112]. LncRNA FENDRR has inhibitory effects on the progression of gastric cancer via miRNA-421/SIRT3/Notch-1 pathway [113]. Furthermore, circFAM160A2 interacted with miRNA-505-3p and SIRT3 to promote mitochondrial stabilization [114]. CircCCDC66 promoted the progression of osteoarthritis by sponging miRNA-3622b-5p and upregulating SIRT3 expression [115].

#### 2.3.4 SIRT4

NcRNAs participate in modulating SIRT4 expression. Several miRNAs can inhibit the expression of SIRT4. Exosomal miRNA-320a derived from human amniotic mesenchymal stem cells downregulated SIRT4 to suppress oxidative stress [116]. Moreover, miRNA-130b-5p and miRNA-424 play a role in the downregulation of SIRT4 [117, 118]. LncRNA MALAT1 attenuated cardiac hypertrophy via the miRNA-93-5p/SIRT4 axis [119], whereas circOMA1 aggravated breast cancer by regulating the miRNA-1276/SIRT4 axis [120].

#### 2.3.5 SIRT5

MiRNAs such as miRNA-19b, miRNA-145-5p, and miRNA-299-3p downregulated SIRT5 expression [121-123], whereas lncRNA SNHG14 and linc01234 upregulated SIRT5 expression through sponging miRNA-656-3p and miRNA-27b-5p, respectively [124, 125]. Moreover, lncRNA APCDD1L-AS1 inhibited autophagic degradation of epidermal growth factor receptors via the miRNA-1322/miRNA-1972/miRNA-324-3p/SIRT5 pathway in lung adenocarcinoma [126]. CircKlhl8 promoted endothelial progenitor cell-mediated diabetic wound healing by sponging miRNA-212-3p and increasing SIRT5 levels [127].

#### 2.3.6 SIRT6

A large amount of evidence indicates that ncRNAs are involved in modulating SIRT6. MiRNAs such as miRNA-34a, miRNA-34c-5p, miRNA-186, and miRNA-654-5p target SIRT6 for silencing [128-131]. Besides, lncRNA SNHG12 alleviated hypertensive vascular endothelial injury via the miRNA-25-3p/SIRT6 pathway [132]. CircITCH, a tumor-suppressive circRNA, attenuated doxorubicin-induced cardiotoxicity by the miRNA-330-5p/SIRT6 axis [133] and improved streptozotocin-induced diabetic renal fibrosis through the miRNA-33a-5p/SIRT6 axis [134].

#### 2.3.7 SIRT7

NcRNAs regulate the expression of SIRT7. Massive studies have identified that miRNAs such as miRNA-125a-5p, miRNA-125b, miRNA-152-3p, and miRNA-770 serve as upstream regulators of SIRT7 by targeting its 3′ UTR [135-137]. Besides, lncRNA AC007207.2 drove the upregulation of SIRT7 by sponging miRNA-1306-5p in osteosarcoma cells [138], while lincRNA p21 elevated SIRT7 levels by sponging miRNA-17-5p in vascular smooth muscle cells. SIRT7 expression was regulated by circRNA ZNF609 and circRNA PVT1 via miRNA-138-5p [139] and miRNA-125b/miRNA-200a sponging [140], respectively.

### 2.4 Post-translational modification of sirtuins

**Figure 4.**
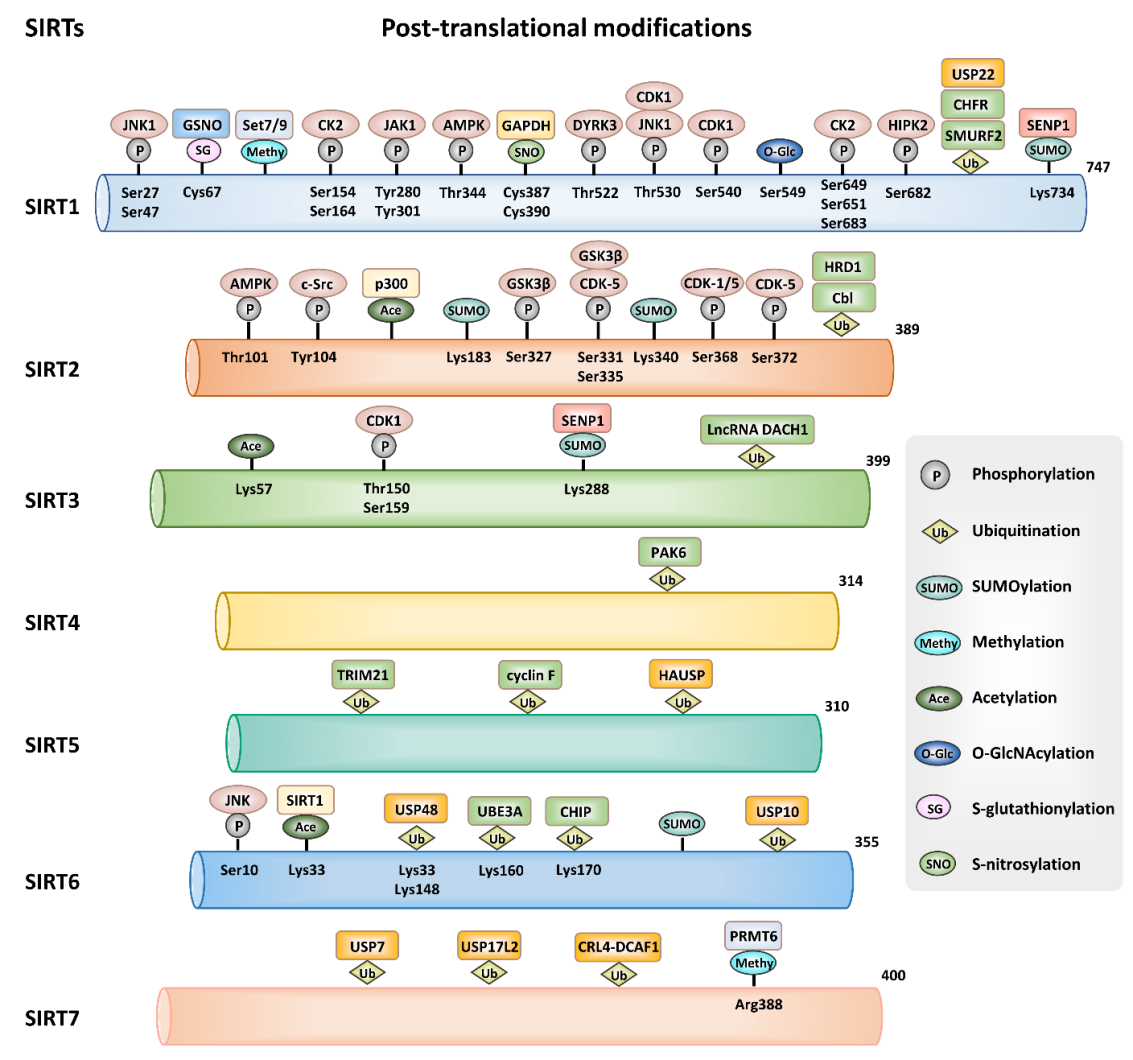
Post-translational modifications of sirtuins. Sirtuins can be affected by post-translational modifications, including phosphorylation, ubiquitination, SUMOylation, methylation, acetylation, O-GlcNAcylation, S-nitrosylation, and S-glutathionylation.

In addition to being regulated at the transcriptional and post-transcriptional levels, sirtuins are also affected by post-translational modifications, including phosphorylation, methylation, acetylation, ubiquitination, SUMOylation, O-GlcNAcylation, S-nitrosylation, and S-glutathionylation (Fig.4).

#### 2.4.1 SIRT1

Post-translational modifications of SIRT1 include ubiquitination [141], SUMOylation [142], phosphorylation [143], methylation [144], O-GlcNAcylation [145], S-nitrosylation [146], and S-glutathionylation [147], which play important roles in regulating SIRT1 function. E3 ubiquitin ligase SMURF2 inhibited the proliferation of colorectal cancer cells and tumor growth through interacting with SIRT1 and mediating its ubiquitination and degradation [141]. SUMOylation of SIRT1 at lysine 734 enhanced its deacetylation ability. Conversely, SUMO-specific peptidase 1 (SENP1)-mediated SIRT1 de-SUMOylation downregulated its enzymatic activity [142]. SIRT1 phosphorylation is mediated by a variety of kinases and is crucial to SIRT1 enzymatic activity. Casein kinase 2 (CK2)-mediated SIRT1 phosphorylation on serine residues 154, 649, 651, and 683 [148] and c-Jun N-terminal kinase 1 (JNK1)-mediated phosphorylation on serine 27, serine 47, and threonine 530 [149] markedly promoted SIRT1 activity and function. In addition, cyclin-dependent kinase 1 (CDK1) phosphorylated SIRT1 on threonine 530 and serine 540 [143]. Wang et al. revealed that Janus kinase 1 (JAK1) catalyzed the phosphorylation of SIRT1 at tyrosine residues 280 and 301 without altering its deacetylase activity [150]. SIRT1 can be O-GlcNAcylated at serine 549 and has cytoprotective effects by increasing its deacetylase activity during stress [145]. Methyltransferase Set7/9 indirectly regulated p53 function by methylating SIRT1 at multiple lysine residues within its N-terminus [144]. Inducible nitric oxide synthase (iNOS) and inflammatory stimuli triggered the S-nitrosylation of SIRT1 and inhibited its enzymatic activity [146, 151]. Glutaredoxin 2 participated in vascular development by removing the S-glutathionylation of SIRT1 [152].

#### 2.4.2 SIRT2

The function of SIRT2 is influenced by post-translational modifications. Cyclin-dependent kinases regulate SIRT2 phosphorylation. CDK-1 can phosphorylate SIRT2 at serine 368 [153], whereas CDK-5 induced SIRT2 phosphorylation at serine residues 331, 335, 368, and 372 [154, 155]. Mass spectrometry confirmed that in the cellular model of PD, SIRT2 was phosphorylated by GSK3β at serine residues 327, 331, and 335 [156]. In addition, AMPK phosphorylated SIRT2 at threonine 101 [157]. SRC proto-oncogene, nonreceptor tyrosine kinase (c-Src) reduced the SIRT2 protein levels via phosphorylation of SIRT2 at tyrosine 104 [158]. Moreover, p300 drove the acetylation of SIRT2 and then reduced the deacetylase activity of SIRT2 [159]. SIRT2-SUMOylation at lysine 183 and 340 plays a crucial role in cellular signal transduction and tumor suppression [160]. Casitas B-lineage lymphoma (Cbl) proteins Cbl-b and c-Cbl promoted the stability and protein expression of SIRT2 via ubiquitination [161]. E3 ubiquitin ligase (HRD1)-mediated SIRT2 ubiquitination and degradation in lung tumorigenesis [162].

#### 2.4.3 SIRT3

At the post-translational level, SIRT3 is affected by phosphorylation, SUMOylation, acetylation, and ubiquitination. CDK1-induced SIRT3 phosphorylation at threonine 150 and serine 159 increased SIRT3 enzymatic activity [57]. SUMOylation negatively regulated SIRT3 enzymatic activity. SENP1-mediated SIRT3 de-SUMOylation at lysine 288 upregulated its deacetylase activity and participated in mitochondrial metabolism [163]. SIRT3 can also be modified by acetylation. In one study, Kwon et al. found that SIRT3 was acetylated at lysine 57 in aged and obese mice [164]. Moreover, berberine [165] and lncRNA DACH1 [166] promoted ubiquitination-mediated SIRT3 degradation by activating the AMPK pathway and increasing intracellular reactive oxygen species (ROS) levels.

#### 2.4.4 SIRT4

Compared to other sirtuins, only a few publications focused on post-translational modifications of SIRT4. For instance, Li et al. found that P21-activated kinase 6 (PAK6) was implicated in mitochondrial apoptosis by promoting ubiquitination-mediated SIRT4 degradation [167]. More extensive studies are required to uncover the modifications underlying SIRT4.

#### 2.4.5 SIRT5

Several researchers have proposed that SIRT5 is regulated by ubiquitination. Yao et al. indicated that tripartite motif-containing protein 21 (TRIM21) interacted with SIRT5 and catalyzed the ubiquitination and degradation of SIRT5. Besides, herpes virus-associated ubiquitin-specific protease (HAUSP) mediated SIRT5 de-ubiquitination and stabilization. In a feedback loop, ubiquitination-induced SIRT5 degradation sustained TRIM21 acetylation at lysine 351 [168]. Moreover, cyclin F-Skp1, CUL1, and the F-box protein family of E3 ubiquitin ligase complex regulated the ubiquitination, abundance, and stability of SIRT5 [169].

#### 2.4.6 SIRT6

The enzymatic activity and protein stability of SIRT6 are modulated by post-translational modifications. As the SIRT6 N-terminus is responsible for its interaction with SIRT1, Meng et al. suggested that SIRT1 mediated the deacetylation of SIRT6 at lysine 33 [170]. Under stress conditions, SIRT6 was phosphorylated by JNK at serine 10. SIRT6 phosphorylation drove its mono-ADP-ribosyltransferase activity on poly(ADP-ribose) polymerase 1 (PARP1) and stimulated the repair of double-strand DNA breaks [171]. Ubiquitin ligase CHIP induced noncanonical ubiquitination of SIRT6 at lysine 170 [172]. Mammalian ubiquitin-specific peptidase 10 (USP10) [173] and USP48 [174] were SIRT6-specific de-ubiquitinases. USP48 was positively regulated by methyltransferase-like 14 (Mettl14)-induced m^6^A modification and promoted SIRT6 de-ubiquitination at lysine 33 and lysine 128. Moreover, SIRT6 ubiquitylation at lysine 160 was mediated by UBE3A [175]. NAD(P)H: quinone oxidoreductase 1 (NQO1) repressed proteasome-mediated degradation of SIRT6 [176]. In addition, SIRT6 can be modified by SUMOylation and decreased its deacetylase activity of histone H3 at lysine 56 [177].

#### 2.4.7 SIRT7

The activity of SIRT7 is affected by methylation and ubiquitination. *In vivo* and *in vitro* studies have revealed that protein arginine methyltransferase 6 (PRMT6) directly interacted with and methylated SIRT7 at arginine 388, leading to the suppression of SIRT7 deacetylase activity [178]. Besides, USP7- [179] and USP17L2-mediated [180] SIRT7 de-ubiquitination alleviated its enzymatic activity, whereas Cullin 4 RING E3 ubiquitin ligase (CRL4)-DCAF1 E3 ligase triggered SIRT7 ubiquitination and degradation [181].

**Figure 5.**
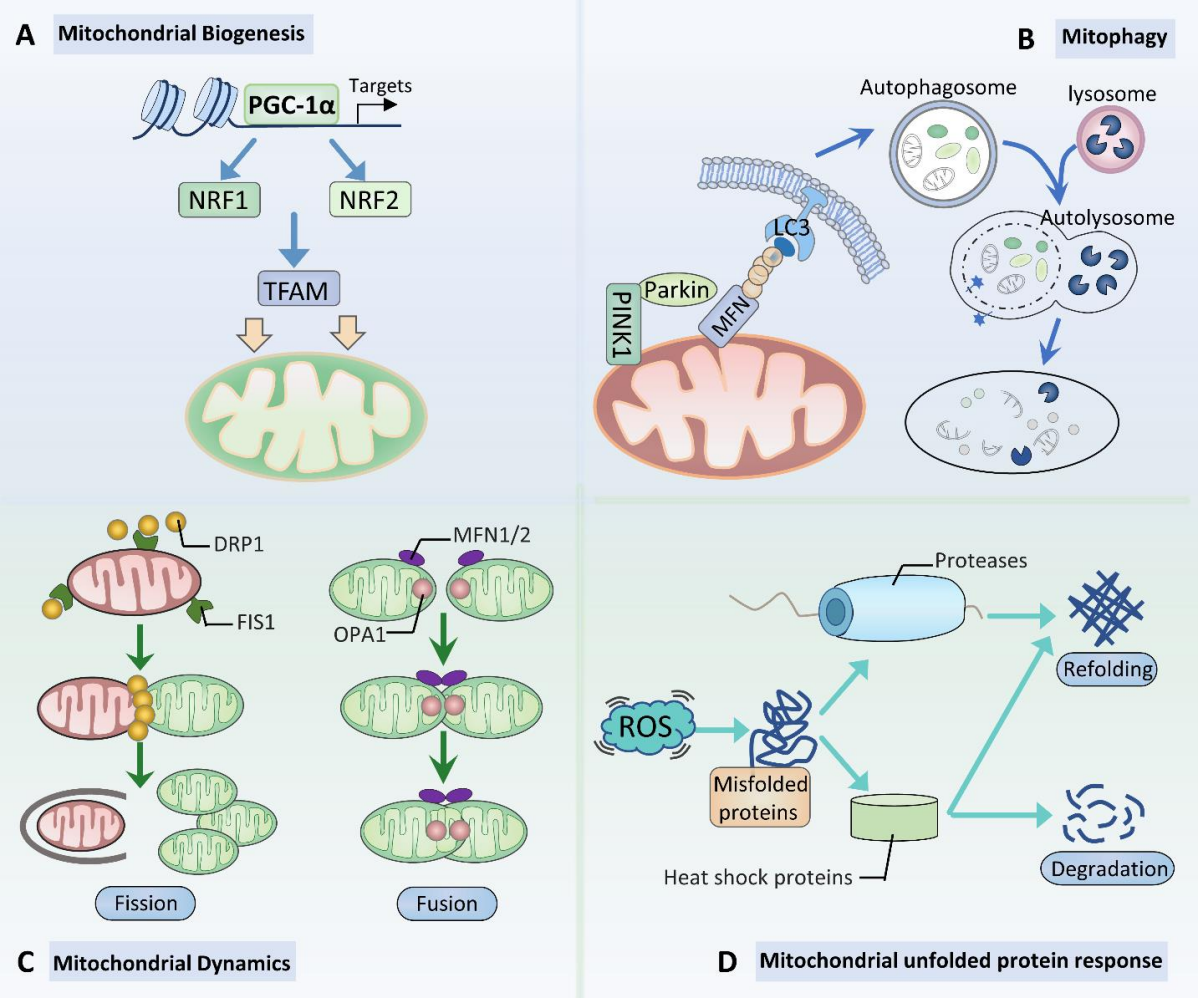
Mitochondrial quality control. (A) Mitochondrial biogenesis. Once activated by either deacetylation or phosphorylation, proliferator-activated receptor γ coactivator 1α (PGC-1α) triggers the activation of the nuclear respiratory factors (NRFs)/mitochondrial transcription factor A (TFAM) pathway, promoting mitochondrial biogenesis. (B) Mitophagy. The phosphatase and tensin homolog-induced putative protein kinase 1 (PINK1)/Parkin-mediated mitophagy pathway is involved in the formation of autophagosomes. (C) Mitochondrial dynamics. Mitochondrial fission is regulated by dynamin-related protein 1 (DRP1) and fission protein 1 (FIS1), whereas mitochondrial fusion is modulated by mitofusin (MFN) 1/2 and optic atrophy 1 (OPA1). (D) Mitochondrial unfolded protein response. Molecular chaperones and proteases mediate the degradation or refolding of misfolded/unfolded mitochondrial proteins.

### 2.5 The NAD/NADH ratio

Because sirtuins are a family of NAD^+^-dependent enzymes, the intracellular level of NAD is key to their enzymatic activities. In addition to their roles in gene expression, NAD^+^ and NADH play significant roles in energy metabolism and mitochondrial function [182]. Using magnetic resonance-based NAD detection *in vivo*, Zhu et al. revealed age-dependent reductions in NAD^+^ levels, NAD^+^/NADH redox potential, total NAD content, and age-dependent increases in intracellular NADH in the brains of healthy subjects [183]. The level of NAD^+^ in the brain decreases with age and negatively affects the activity of sirtuins [184]. Many studies have confirmed that the NAD^+^/NADH ratio is highly related to the activity of sirtuins in diverse pathophysiological processes. Crucially, exogenously introduced NAD^+^ increased sirtuin activity [185]. Nicotinamide mononucleotide adenylyltransferase (Nmnat) is a crucial enzyme in NAD biosynthesis. Cai et al. revealed that Nmnat2 ameliorated angiotensin II-induced cardiac hypertrophy by maintaining NAD levels and activating SIRT6 [186].

A broad range of evidence has described the function and regulatory mechanisms of sirtuins under different physiological conditions. Indeed, understanding of the functions of sirtuins is improving.

## 3. Mitochondrial quality control in the pathogenesis of neurodegenerative diseases

Aging neurons frequently show evidence of mitochondrial dysfunction, which is associated with the pathogenesis of neurodegeneration [6]. Recently, the role of mitochondrial quality control in neurodegenerative diseases has received increased attention. Advances in mitochondrial quality control, including mitochondrial biogenesis, mitophagy, mitochondrial fusion and fission, and mtUPR, provide attractive approaches to the prevention and treatment of neurodegenerative diseases (Fig. 5).

### 3.1 Mitochondrial biogenesis in neurodegenerative diseases

Mitochondrial biogenesis, the process by which new mitochondria are generated from existing mitochondria, is mediated by PGC-1α [187]. PGC-1α, once activated by either deacetylation or phosphorylation, triggers the activation of the NRFs/mitochondrial transcription factor A (TFAM) signaling pathway, subsequently favoring mitochondrial biogenesis by the production of mitochondrial DNA (mtDNA) and proteins [188]. Mitochondrial biosynthesis is required to sustain mitochondrial respiration, maintain mitochondrial redox balance, and prevent mitochondrial apoptosis. Mitochondrial biogenesis is crucial to maintaining mitochondrial homeostasis.

Impaired mitochondrial biogenesis is associated with the development of AD. In one study, pyramidal neurons in the AD hippocampus showed a significant reduction in mtDNA copy numbers and disruption of mitochondrial biogenesis transcriptome signaling compared to the control hippocampus [189]. Besides, PGC-1α mRNA expression was remarkably decreased with the deterioration of dementia in the AD brain and was negatively correlated with amyloid beta (Aβ) and Tau pathology [190]. Notably, Sheng et al. demonstrated that the expression of PGC-1α, NRF1, NRF2, and TFAM in the AD hippocampus was decreased. *In vitro* studies have shown that restoration of PGC-1α expression has a salvage effect on mitochondrial and neuronal function in AD cellular models [191]. Moreover, PGC-1α modulates β-site amyloid precursor protein cleaving enzyme 1 (BACE1) and synergies with SIRT1-mediated PPARγ deacetylation constitute a key mechanism regulating Aβ production in AD [192].

Regulation of mitochondrial biogenesis is a promising therapeutic target for PD [193]. PGC-1α single nucleotide polymorphisms rs6821591 and rs2970848 were relative to the risk of onset of PD [194]. Besides, inhibition of PGC-1α activity is a primary cause of *PINK1* and *Parkin* mutations-induced family PD [195]. PGC-1α was reduced in the leukocytes, substantia nigra, and brain. Another study revealed that mRNA levels of mitochondrial biogenesis activator genes such as PGC-1α and NRF1 and repressor genes such as ZFP746 and Mybbp1a were evidently decreased at all stages in MPTP-induced PD mouse models [196]. Ye et al. demonstrated that PGC-1α overexpression suppressed MPP^+^-induced mitochondrial dysfunction in SHSY5Y cells and showed a neuroprotective role in HD via the ERRα, PPARγ, and NRF1 pathways [197].

Defective mitochondrial biogenesis and impaired PGC-1α signaling pathway are associated with striatal vulnerability in HD [198]. The mRNA levels of PGC-1α in the striatum were decreased in early HD patients [199]. Mutant huntingtin (mTTT) inhibited PGC-1α gene transcription by interfering with the cAMP response element-binding (CREB)/TAF4-dependent transcriptional pathway. Overexpression of PGC-1α in the striatum partially reversed mTTT-induced neurotoxicity in the striatal neurons [199]. Additionally, the PGC-1α downstream transcription factors NRF1 and TFAM have been proposed as genetic modifiers of HD [200].

### 3.2 Mitophagy in neurodegenerative diseases

Mitophagy is a process of selective elimination of mitochondrial dysfunction through the autophagy-lysosomal pathway [201]. One important mitophagy pathway in mammals relies on phosphatase and tensin homolog-induced putative protein kinase 1 (PINK1) and the E3 ubiquitin ligase Parkin [202]. Impaired mitophagy leads to the accumulation of damaged mitochondria, which ultimately causes the progression of neurodegenerative diseases.

Affected neurons in AD suffer from mitochondrial dysfunction and early-onset bioenergetic defects, which contribute to Aβ and Tau pathology. Compelling evidence suggested that mitophagy was impaired in AD, resulting in the accumulation of dysfunctional mitochondria [203]. In cellular and animal AD models, energy deficiency and oxidative stress drove Aβ and Tau accumulation, leading to synaptic and cognitive dysfunction, which in turn impinged on mitophagy. On the one hand, overexpression of phosphorylated Tau alleviated mitophagy through increasing mitochondrial membrane potential and decreasing PINK1 and Parkin levels [204]. On the other hand, elevated mitophagy eliminated Tau hyperphosphorylation and Aβ pathology in neuronal cells and reversed memory impairment in animal models of AD [205, 206]. Therefore, stimulating or promoting mitophagy may block the pathological process of AD and be a potential strategy for AD treatment.

The critical role of mitophagy in the pathogenesis of PD highlights the potential therapeutic application of mitophagy modulation. Many PD-associated genes are related to mitophagy defects. Loss of PINK1 or Parkin function inhibited mitophagy, leading to mitochondrial dysfunction and loss of dopaminergic neurons [207]. In addition, *LRRK2* mutant is the most common monogenic cause of PD. RAB10 is a substrate of LRRK2 kinase that accumulates on depolarized mitochondria in a PINK1- and Parkin-like manner. *LRRK2* mutation disrupted depolarization-induced mitophagy through repressing mitochondrial accumulation of RAB10 [208].

HD is a common neurodegenerative disease associated with abnormal polyglutamine (PolyQ) expansion-induced misfolding proteins. Mitochondrial glyceraldehyde-3-phosphate dehydrogenase (GAPDH) interacted with expanded PolyQ tracts and disturbed GAPDH-mediated mitophagy, resulting in the accumulation of damaged mitochondria and cell death [209]. Of note, mHTT impairs mitophagy in HD [210]. Huntingtin protein acted as a scaffold protein of autophagy protein 11 (ATG11) and was implicated in selective autophagy [211]. Further studies showed that mitophagy was altered in the presence of mHTT through a reduction in the ability of mitochondria to target autophagosomes. Khalil et al. found that overexpression of PINK1 in HD striatal cells could partially restore mitophagy [212].

### 3.3 Mitochondrial dynamics in neurodegenerative diseases

Mitochondrial fusion and fission are crucial in mammals, and their impairment is strongly associated with a broad range of diseases, especially neurodegenerative diseases [213]. Mitochondrial fusion is a multistep process that includes outer and inner mitochondrial membrane fusion. Mechanistically, outer mitochondrial membrane fusion is mediated by mitochondria-related proteins mitofusin1 (MFN1) and MFN2, whereas inner mitochondrial membrane fusion is regulated by optic atrophy 1 (OPA1) [214]. Mitochondrial fission proteins include dynamin-related protein 1 (DRP1) and mitochondrial fission protein 1 (FIS1) [215]. In addition, mitochondrial fission is also mediated by other proteins, including mitochondrial fission factor (MFF), mitochondrial dynamic protein 49 (MiD49), and MiD51 [213].

Imbalance of mitochondrial fission and fusion is a key driver of mitochondrial dysfunction and neuronal injury in the AD brain [216]. Immunoblot analysis showed that the levels of DRP1, OPA1, MFN1, and MFN2 were dramatically decreased in AD, whereas the levels of FIS1 were vastly increased [216]. DRP1 interacted with Aβ and phosphorylated Tau, resulting in a series of neurotoxic events, including excessive mitochondrial fragmentation and ROS production, and mitochondrial bioenergetics and synaptic defects, ultimately causing neuronal and cognitive impairment [217]. Baek et al. revealed that DRP1 inhibition attenuated mitochondrial fragmentation, alleviated mitochondrial membrane potential loss, and ameliorated ROS generation in Aβ-treated neurons. Moreover, suppression of DRP1 rescued learning and memory deficits in AD mice and prevented mitochondrial fragmentation, BACE1 expression, and Aβ deposition in the AD brain [218]. Indeed, Aβ-induced nitric oxide generation was involved in mitochondrial fission, synaptic loss, and neuronal dysfunction partly through DRP1 S-nitrosylation, whereas DRP1 nitrosylation prevention eliminated these deleterious effects [219]. Furthermore, Aβ-induced DRP1 phosphorylation triggered excessive mitochondrial fission, ultimately leading to neuronal apoptosis [220]. Therefore, targeting mitochondrial fission may be a promising therapeutic method for AD.

DRP1-mediated aberrant mitochondrial fission contributes to PD [221]. Elevated DRP1 promoted mitochondrial fission and dopaminergic neuronal apoptosis, whereas suppression of DRP1 reversed aberrant mitochondrial fission and attenuated cell apoptosis [222]. Additionally, LRRK2 participated in regulating mitochondrial dynamics by enhancing and interacting with DRP1 [223]. PD-related genes such as *PINK1* and *Parkin* were also found to exhibit vital roles in the modulation of mitochondrial dynamics [224]. Overexpression of α-synuclein contributes to mitochondrial fragmentation [225].

In HD patients, the mRNA and protein levels of DRP1 and FIS1 were increased, whereas the expression of MFN1 and MFN2 was decreased, which was consistent with the elevated mitochondrial fragmentation in cortical neurons [226]. Shirendeb et al. reported that mHTT interacted with DRP1 to enhance the enzymatic activity of GTPase DRP1 in postmortem HD brains and primary neurons from transgenic BACHD mice, worsening abnormal mitochondrial dynamics and ultimately resulting in defective axonal transport and synaptic degeneration in HD [227]. Moreover, mHTT promoted post-translational modifications and enhanced DRP1 activity and mitochondrial fission [228]. Inhibition of DRP1 overactivation downregulated neuropathological and behavioral deficits in zQ175 knock-in HD mouse models [229].

### 3.4 mtUPR in neurodegenerative diseases

mtUPR is a stress-protective response that ameliorates mitochondrial damage and maintains mitochondrial proteostasis by repairing or eliminating misfolded proteins [230]. Mitochondrial chaperones, such as heat shock protein 9 (HSP9), HSP10, HSP60, and HSP70, are implicated in helping restore misfolded proteins to normal conformations and improving the correct folding of newly synthesized proteins. Proteases, including CLP protease proteolytic (CLPP), YME1-like 1 ATPase (YME1L1), and Lon protease, are involved in degrading unwanted proteins [231].

AD is characterized by the accumulation of two insoluble protein aggregates, Aβ plaques and tau neurofibrillary tangles. AD is marked by an excess of faulty or unfolded proteins, which activates UPR [232]. *In vivo* and *in vitro* studies have shown that mtUPR was activated in the AD mice brain and Aβ_25-35_-treated SHSY5Y cells. Inhibition of mtUPR intensified Aβ_25-35_-induced cytotoxic effects on SHSY5Y cells, suggesting that mtUPR plays a protective role in the progression of AD [233]. Sorrentino et al. reported that mtUPR was essential for maintaining mitochondrial proteostasis. Moreover, increased mitochondrial proteostasis inhibited Aβ aggregation in cells, worms, and mouse models of AD [234]. The expression of mtUPR-related genes, including HSP60, CLPP, and YME1L1, was significantly increased by 40% to 60% in individuals with sporadic AD and by approximately 70% to 90% in familial AD subjects compared to cognitively intact controls [235]. Promoting the activation of mtUPR has emerged as an effective therapeutic modality for AD.

mtUPR is also implicated in PD. In *C. elegans*, mitochondrial damage causes the nuclear translocation of the transcription factor ATFS-1 to activate mtUPR [236]. PINK1 mutant activated ATFS-1-dependent mtUPR and promoted dopamine neuron survival in PD [237]. Furthermore, mtUPR overactivation increased α-synuclein-induced dopaminergic neurotoxicity, whereas ATFS-1 deficiency attenuated α-synuclein-related neurotoxicity [236]. In a nutshell, mtUPR activation suppressed PD by enhancing the survival of dopaminergic neurons, whereas excessive mtUPR activation was detrimental to PD.

Berendzen et al. found that polyQ-40 bound to mitochondria and elicited mtUPR in *C. elegans* neurons, hinting at an association between mtUPR and HD [238]. The mitochondrial transporter ABCB10 implicated in the mtUPR pathway was decreased in fibroblasts from HD patients and in striatal cells from HD mice. mHTT inhibited mtUPR by impairing the stability of ABCB10 mRNA [239]. Additionally, the levels of HSP60 and CLPP were significantly reduced in the ABCB10-deficient HD model. ABCB10 was involved in modulating CHOP, a transcription factor of HSP60 and CLPP. These findings suggest that moderate activation of mtUPR may have a therapeutic effect on HD.

## 4. Roles of sirtuins in mitochondrial quality control

**Figure 6.**
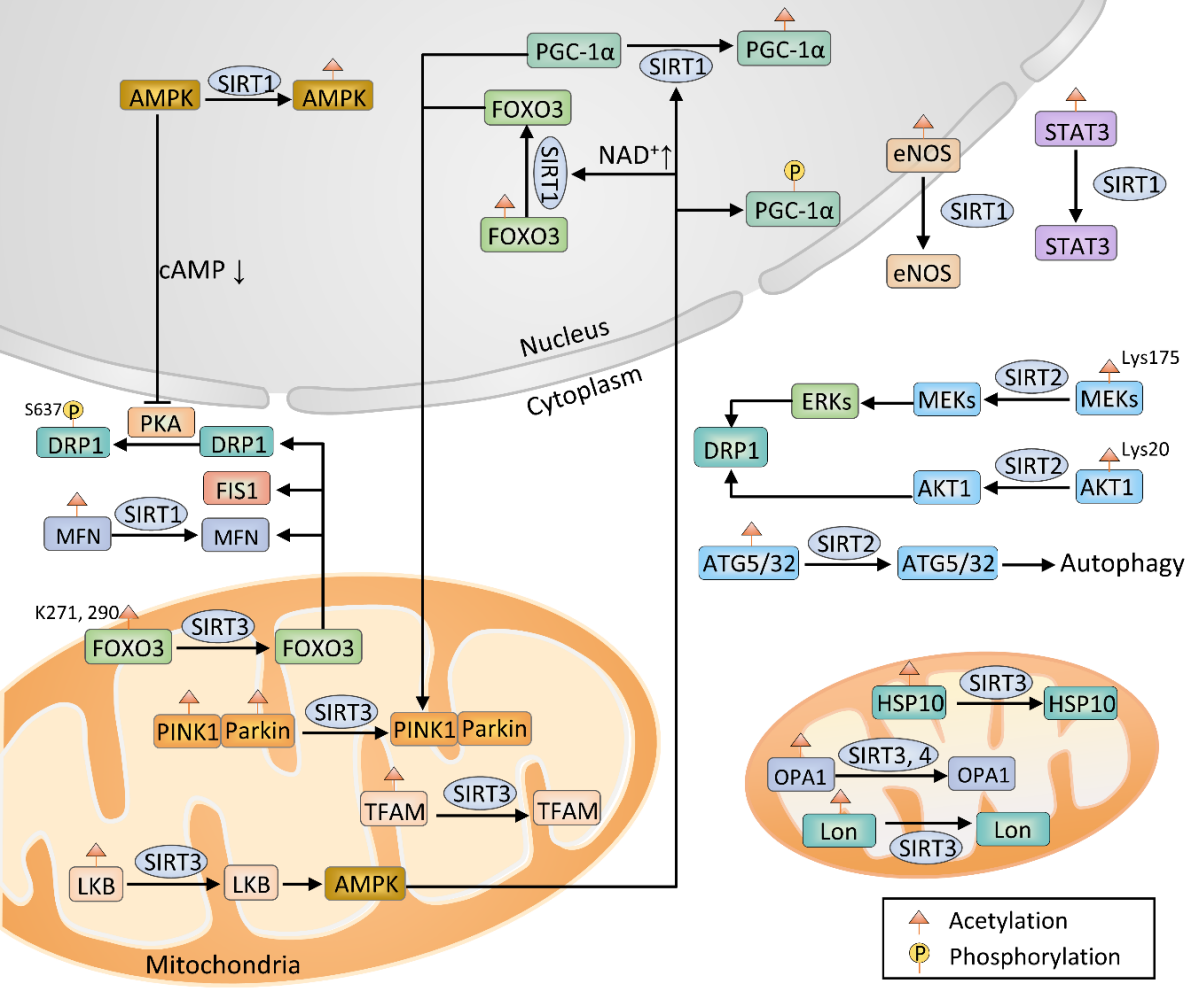
Sirtuin-mediated mitochondrial quality control in neurodegeneration. SIRT1 catalyzes the deacetylation of proliferator-activated receptor γ coactivator 1α (PGC-1α), AMP-activated protein kinase (AMPK), forkhead box class O3 (FOXO3), mitofusin (MFN), signal transducer and activator of transcription 3 (STAT3), and endothelial nitric oxide synthase (eNOS) and participates in mitochondrial quality control. PINK1/Parkin pathway-mediated mitophagy is activated by either the SIRT1/FOXO3 axis or SIRT3. Additionally, SIRT2 is involved in mitochondrial fission by regulating DRP1 via the SIRT2/mitogen-activated protein kinase kinase-1 (MEK1)/extracellular signal-regulated kinase (ERK)/dynamin-related protein 1 (DRP1) and SIRT2/serine/threonine-protein kinase AKT1/DRP1 pathways. Autophagy protein (ATG) 5/32-mediated mitophagy is regulated by SIRT2. SIRT3 deacetylates HSP10 and Lon proteases and regulates mitochondrial unfolded protein response (mtUPR). Liver kinase B (LKB), mitochondrial transcription factor A (TFAM), FOXO3, and optic atrophy type 1 (OPA1) have been identified as SIRT3 substrates. Additionally, SIRT4 modulates mitochondrial dynamics by regulating OPA1.

Sirtuins are involved in almost all aspects of mitochondrial metabolism and homeostasis, protecting mitochondria from various types of damage. With a deeper understanding of the molecular mechanisms of the acetylation of multiple mitochondrial proteins involved in stress and metabolism, sirtuins are implicated in regulating mitochondrial quality control, including mitochondrial biogenesis, mitophagy, mitochondrial fusion and fission, and mtUPR (Fig. 6).

### 4.1 Sirtuins in mitochondrial biogenesis

There is converging evidence that sirtuins play a significant role in regulating mitochondrial biogenesis. SIRT1 is heavily involved in mitochondrial biogenesis and longevity. The SIRT1/PGC-1α signaling pathway showed an endogenous neuroprotective role in confronting epileptic seizure-induced neuronal cell damage [240]. In addition, SIRT1 indirectly regulated mitochondrial biogenesis by upregulating endothelial nitric oxide synthase or catalyzing signal transducer and activator of transcription 3 (STAT3) deacetylation [241]. SIRT2 plays various roles in cellular metabolism, especially during aging. Mitochondrial dysfunction and neurite outgrowth suppression were mediated by high d-glucose concentration-induced increases in polyol pathway activity and further depletion of SIRT2 [242]. Furthermore, SIRT2-knockout mice showed mitochondrial failure, redox imbalance, and energy depletion [243]. SIRT3 overexpression elicited activation of the AMPK signaling pathway via the deacetylation of liver kinase B1 (LKB1), promoting mitochondrial biogenesis and mitochondrial turnover [244]. Activated AMPK promoted SIRT1 activity by enhancing cellular NAD^+^ levels, leading to the deacetylation of SIRT1 downstream targets [245]. The PGC-1α/SIRT3/UCP2 axis was participated in improving mitochondrial biogenesis, dynamics, and function [246]. SIRT3 triggered the deacetylation and activation of FOXO3, subsequently upregulating the expression of PGC-1α and TFAM [247]. Moreover, SIRT3 promoted mitochondrial biogenesis through enhancing the content of mtDNA and the deacetylation of TFAM [248]. SIRT4 has been linked to mitochondria-derived signaling cascades by the regulation of AMPK and PGC-1α [249]. Moreover, PGC-1α overexpression and food withdrawal increased the levels of SIRT5 mRNA. Activated AMPK decreased the expression of SIRT5 mRNA. Overexpression of SIRT5 enhanced oxygen consumption and ATP generation but did not affect mitochondrial biogenesis [74]. Similarly, SIRT6 regulated mitochondrial biogenesis by increasing the activities of AMPK and PGC-1α or reducing the activity of Sox6 [250, 251]. Furthermore, arginine 388 methylation of SIRT7 modulated mitochondrial respiration and mitochondrial biogenesis [178]. Nevertheless, the molecular mechanisms of SIRT4, SIRT5, SIRT6, and SIRT7 underlying mitochondrial biogenesis in neurodegenerative diseases remain elusive.

### 4.2 Sirtuins in mitophagy

Sirtuins play a critical role in the regulation of mitophagy via acetylation. SIRT1 signaling cascade mediated mitophagy and PINK1/Parkin mitochondrial translocation in human neuroblastoma SHSY5Y cells through activation of the PGC-1α pathways [252]. *In vitro* studies have confirmed that the levels of autophagy markers light chain 3B (LC3B) and Beclin1 were significantly reduced and the expression of p62 was pronouncedly increased after pretreatment with SIRT1 inhibitor EX527, indicating that the inactivation of SIRT1 impeded mitophagy. SIRT1-mediated PINK1/Parkin-dependent mitophagy has neuroprotective effects [253]. Besides, activated SIRT1 promoted mitophagy and ameliorated mitochondrial dysfunction by activating the FOXO1/3/PINK1/parkin signaling pathway [254]. Chang et al. found the possible function of miRNA-302 in improving mitophagy and preserving mitochondrial function through activation of the SIRT1/AMPK/PGC-1α pathway [255]. SIRT2 appears to have unique mitochondrial targets and directs the relevant components of mitophagy. SIRT2 overactivation drove the dysregulation of autophagy and mitophagy. Elevated SIRT2 levels and decreased tubulin lysine 40 acetylation were found in the AD brain [256]. Intriguingly, microtubule assembly was not altered in MPP^+^-treated SIRT2-knockout mice neurons, which maintained normal autophagic flux. The inhibition of SIRT2 enhanced the acetylation of α-tubulin and accelerated the trafficking and clearance of misfolded protein [257]. During aging, increased mitophagy activity regulated by the SIRT2/ATG32 axis was a significant phenomenon associated with α-synuclein-induced toxicity [258]. Besides, mitophagy was impaired in SIRT2-deficient neurons due to the acetylation of ATG5, an E3 ubiquitin ligase [259]. SIRT3 facilitated mitophagy by directly deacetylating PINK1 and Parkin and indirectly regulating PINK1 via the SIRT3/FOXO3 pathway [260]. SIRT3 suppression reversed mitophagy induction through reducing the levels of LC3-II/LC3-I, p62, FOXO3α, and BINP3 [261]. One study confirmed that SIRT3-mediated deacetylation and activation of FOXO3 promoted the expression of LC3, Nix, and BNIP3. The expression of LC3, Nix, and BNIP3 was decreased in SIRT3-knockdown cells [247]. Furthermore, activated SIRT4 and SIRT5 alleviated mitophagy, whereas activated SIRT6 promoted mitophagy. Lang et al. reported that SIRT4 emerged as a stress-triggered factor and exhibited a role in reducing mitophagy, leading to mitochondrial dysfunction and mitochondrial quality control impairment [262]. Autophagy and mitophagy were enhanced in SIRT5-deficient cells and decreased in SIRT5-overexpressed cells [263]. During starvation, SIRT5 deletion enhanced mitophagy by attenuating mitochondrial elongation [264]. Conversely, SIRT1-mediated activation of the SIRT6/AMPK signaling pathway increased mitophagy and sustained mitochondrial function through intensifying PINK1/Parkin and the LC3II/LC3I ratio [265]. No relevant studies on the relationship between SIRT7 and mitophagy have been reported.

### 4.3 Sirtuins in mitochondrial dynamics

Increasing evidence has revealed the primary role of sirtuins in mitochondrial fusion and fission dynamics. SIRT1 was associated with mitochondrial fusion and fission. It promoted mitochondrial elongation through MFN1 deacetylation and accumulation [266]. Activated SIRT1 induced mitochondrial fission by the accumulation of FIS1 and activation of DRP1 via the SIRT1/AMPK/protein kinase A (PKA)/DRP1 pathway. Nicotinamide (NAM)-induced SIRT1 activation leads to mitochondrial fission, at least partly by inhibiting DRP1 phosphorylation to activate DRP1. Mechanistically, activated SIRT1 triggered AMPK activation, which subsequently reduced cellular cAMP levels and repressed the activity of PKA [267]. In addition, after inhibiting Ca^2+^ transfer to mitochondria, SIRT1 works collaboratively with AMPK to promote mitochondrial fragmentation through the deacetylation of cortactin and actin cytoskeleton [268]. SIRT2 inhibition enhanced mitochondrial fission through activating DRP1. SIRT2 modulated mitochondrial dynamics via two axes, SIRT2/mitogen-activated protein kinase kinase-1 (MEK1)/extracellular signal-regulated kinase (ERK)/DRP1 axis and SIRT2/serine/threonine-protein kinase AKT1/DRP1 axis [269]. SIRT3 affected mitochondrial dynamics by interacting with OPA1 and FOXO3. It induced the deacetylation of OPA1 and upregulated its GTPase activity, promoting the preservation of mitochondrial networking [270]. Likewise, SIRT3-mediated FOXO3 deacetylation at K271 and K290 enhanced the expression of MFN2, DRP1, and FIS1 [271]. The SIRT4-OPA1 signaling cascade was associated with mitochondrial quality control. Elevated SIRT4 interacted with and stabilized OPA1, promoting mitochondrial fusion in an enzyme-dependent manner and counteracting mitochondrial fission and mitophagy [262]. Moreover, SIRT5 regulated mitochondrial dynamics. Expression of MiD51 and FIS1 was increased in SIRT5-deletion mouse embryonic fibroblasts, resulting in mitochondrial fission [264]. However, the roles of SIRT6 and SIRT7 in mitochondrial fusion and fission remain obscure.

### 4.4 Sirtuins in mtUPR

The NAD^+^/sirtuin pathway regulates lifespan through activation of mtUPR and FOXO signaling [272]. In one study, pretreatment with the SIRT1 activator resveratrol improved postoperative learning and memory impairment in aged mice and significantly reduced the expression of UPR-related proteins and inflammatory molecules in the hippocampus [273]. Another study found that the expression and activity of SIRT3 in the hippocampus were markedly increased after treatment with honokiol, accompanied by activation of mtUPR [274]. Manganese superoxide dismutase (MnSOD), HSP10, and Lon protease have been identified as STRT3 substrates [275-277]. The relationship between sirtuins and mtUPR is still obscure.

These emerging data support the potential applications of sirtuins in regulating mitochondrial quality control and mitochondrial biology, hinting at the involvement of sirtuins in neurodegenerative diseases and providing promising therapeutic targets for neurodegenerative diseases. More extensive studies, however, are required to uncover the underlying mechanisms and roles of diverse sirtuins in mitochondrial dysfunction.

## 5. Roles of sirtuin-mediated mitochondrial quality control in neurodegenerative diseases

Mitochondrial dysfunction is a key and early contributor to neurodegenerative diseases [278]. Sirtuins have a highly regulatory effect on mitochondrial function, making them exciting targets for the treatment of neurodegenerative diseases. Emerging studies have shown the role of sirtuins in neurodegeneration.

### 5.1 AD

AD is the most common neurodegenerative disorder. The pathology of AD is characterized by synaptic loss and cognitive decline due to the accumulation of Aβ and p-Tau neurofibrillary tangles in the brain [279]. There is particularly strong data showing that Aβ was involved in regulating mitochondrial function and neuronal damage through sirtuins. Aβ reduced SIRT1 and its downstream signaling, leading to increased intracellular ROS accumulation and mitochondrial dysfunction [280].

Rashmita et al. found that serum levels of SIRT1, SIRT3, and SIRT6 were remarkably reduced in AD subjects compared with mild cognitive impairment subjects and geriatric controls. In contrast, there were no significant differences in the expression of SIRT2, SIRT4, SIRT5, and SIRT7 among the three groups [281]. Reciprocally, another study found that the expression of SIRT5 increased as AD progressed [282]. SIRT1 suppressed Aβ generation via biased amyloid precursor protein processing toward the non-amyloidogenic pathway. Decreased SIRT3 levels were highly correlated with cerebral cortical Aβ pathology in AD patients. By preserving mitochondrial function, SIRT3 enhanced the functionality of the GABAergic interneuron and ameliorated neuronal network hyperactivity through inhibiting Aβ-associated dysfunction and degeneration in AppPs1 AD mice [283]. Conversely, activated SIRT2 contributes to mitophagy dysfunction. Silva et al. observed elevated SIRT2 levels and decreased tubulin acetylation in cells containing AD patient mtDNA and AD brains. The lack of SIRT2 improved mitophagy and recovered microtubule stabilization, promoting Aβ elimination and neuronal cell survival [256]. Besides, SIRT5 inhibited neurotrophic pathways and Aβ accumulation in AD through promoting autophagy [284]. SIRT6 plays a role in maintaining genomic stability in the brain, and its loss could potentially cause toxic Tau stability and phosphorylation [285]. These findings indicate a close association between sirtuin-mediated mitochondrial quality control and AD.

### 5.2 PD

PD is the second most common neurodegenerative disease (after AD), with an average age-standardized annual incidence of 160 cases per 100,000 people age 65 or older in high-income countries [286]. Mitochondrial dysfunction contributes to the occurrence and development of sporadic and familial PD. Numerous lines of evidence indicated a bidirectional relationship between α-synuclein and mitochondrial dysfunction [287]. In addition, most PD-related genes, such as *PINK1*, *SNCA*, *LRRK2*, and *CHCHD2*, are implicated in the modulation of mitochondrial homeostasis [202].

Rita et al. found a decreased SIRT1 mRNA level, an increased SIRT6 mRNA level, and an unchanged SIRT2 mRNA level in PD patients in comparison to controls [288]. Emerging research supports the notion that upregulation of SIRT1, SIRT3, and SIRT5 confers neuroprotection against PD. SIRT6 was upregulated in the PD brain and exhibited a proinflammatory and pathogenic role in PD [289]. SIRT1 modulated α-synuclein clearance and phosphorylation in PD through the modulation of mitochondrial function, mitophagy, and heat shock factor 1 deacetylation, decreasing α-synuclein accumulation [290]. Additionally, α-synuclein induced mitochondrial dysfunction via a SIRT3-dependent pathway. α-synuclein interacted with mitochondria, suppressing mitochondrial biogenesis and reducing SIRT3 protein levels. A reduction in SIRT3 levels was accompanied by decreased phosphorylation of AMPK and CREB and increased phosphorylation of DRP1. Besides, treatment with the AMPK agonist 5-aminoimidazole-4-carboxamide-1-β-d-ribofuranoside upregulated SIRT3 expression and reduced α-synuclein generation [291]. SIRT3 overexpression has shown beneficial effects on neuron-saving by stabilizing mitochondrial biogenetics in PD rat models. Pharmacological elevation of SIRT3 levels decreased α-synuclein oligomers and improved mitochondrial biogenesis against α-synuclein-induced mitochondrial dysfunction [291]. SIRT5 attenuated MPTP-induced nigrostriatal dopaminergic neuron degeneration through increasing SOD2 levels and mitochondrial antioxidant capacity [292]. Divertingly, there are conflicting reports on the role of SIRT2 in PD. In one study, SIRT2-knockout mice reduced α-synuclein toxicity and decreased MPTP-induced dopaminergic cell damage [293]. However, SIRT2 inhibition enhanced α-synuclein aggregation and promoted neural cell death [294]. In another study, SIRT6 overexpression developed more severe pathology, whereas SIRT6 knockout mice were protected from MPTP-induced PD [289]. These findings support the claim that sirtuin-mediated mitochondrial quality control is a key mechanism in PD.

### 5.3 HD

HD is an autosomal dominant, progressive neurodegenerative disease. Huntingtin, the mutant protein in HD, is strongly associated with cellular damage, including mitochondrial dysfunction, synaptic disruption, and decreased axonal transport rates [6]. mHTT is involved in regulating mitochondrial dysfunction. Additionally, mHTT can directly interact with mitochondrial proteins and act as a translocase of the inner membrane 23 (TIM23), disrupting mitochondrial proteostasis and promoting ROS production and HD progression [295].

Baldo et al. showed that SIRT1 was enhanced in the striatum and cerebral cortex, SIRT2 was only increased in the striatum, and SIRT3 was not changed in HD patients [296]. SIRT1 protected neurons from the toxicity of mHTT. Data on the role of SIRT2 in HD are mixed. The accumulation of mHTT increased sterols in neurons, whereas inhibition of SIRT2 decreased sterols through inhibiting the nuclear trafficking of SREBP-2 [297]. In contrast, Bobrowska et al. found that a reduction in SIRT2 did not affect tubulin acetylation in the brain or the development of HD [298]. In addition, the expression of mHTT inhibited SIRT3 deacetylase activity, further leading to a decrease in intracellular NAD^+^ levels and mitochondrial biogenesis [299]. SIRT3 provided neuroprotection in HD through modulating oxidative stress and mitochondrial dynamics. For example, ε-viniferin increased SIRT3-mediated FOXO3 deacetylation and alleviated rotenone-induced cell apoptosis by maintaining mitochondrial homeostasis and reducing oxidative stress [300]. Compelling evidence suggested the potential therapeutic implications of sirtuins for neurodegenerative disorders.

## 6. Pharmacological exploitation of sirtuins for therapeutic purposes

Given their important role in regulating mitochondrial quality control, it is not surprising that sirtuins are involved in the regulation of neurodegeneration. Therefore, sirtuins have been proposed as promising therapeutic targets for neurodegenerative diseases. Mitochondrial dysfunction occurring in neurodegenerative disorders can be partially reversed by exercise training, calorie restriction, and sirtuin modulators to regulate sirtuin activity.

### 6.1 Exercise training and calorie restriction

Exercise training and calorie restriction are beneficial for controlling disorders associated with aging, especially neurodegenerative diseases. Exercise can alleviate the progression of cognitive impairment in the elderly [301]. Physical exercise has a protective effect on the vulnerability of dopaminergic neurons and the progression of PD. An intriguing study found a reduction in mRNA levels of SIRT1 and SIRT3 in the nigral region of sedentary aged rats compared to young rats. In contrast, treadmill running significantly increased mRNA and protein levels of SIRT1 and SIRT3 in the nigral region of aged rats but not in young rats [302]. Elevated SIRT1 levels induced by treadmill exercise facilitated the non-amyloidogenic pathway and suppressed the amyloidogenic pathway through increasing ADAM-10 and upregulating PGC-1α, respectively [303]. Exercise inhibited α-synuclein levels in a PD mouse model via SIRT1 [304]. In addition, physical exercise efficiently protected against the development of AD by mitigating mitochondrial dysfunction via SIRT1/FOXO1/3/PINK1/parkin-related mitophagy [254]. Calorie restriction enhanced the life span and ameliorated brain health. Ma et al. reported that the neuroprotective role of calorie restriction was partially ascribable to the SIRT1/mTOR pathway and SIRT1-mediated prevention of AD-type Aβ neuropathology [305].

### 6.2 Drug discovery and the development of sirtuin modulators

In addition to physical exercise and calorie restriction, a great deal of research has shown the potential therapeutic applications of sirtuin modulators in the prevention and treatment of neurodegenerative diseases. Some of these compounds have a positive effect on improving mitochondrial quality control.

#### 6.2.1 Cell and animal models

Natural phytochemical compounds modulate sirtuin activity. Resveratrol is the most well-known activator of SIRT1 [306]. It is beneficial to mitochondrial biogenesis and prevents metabolic decline. An intriguing study found enhanced mitochondrial function and biogenesis in mice treated with moderate doses of resveratrol, while SIRT1 knockout mice did not have these benefits [307]. Another study reported that resveratrol activated SIRT1-relevant mitochondrial biogenesis through the SIRT1/PGC-1α/NRF1/TFAM pathway, alleviating sodium fluoride-induced neurotoxicity [308]. Furthermore, sildenafil inhibited the expression of β-secretase 1 and the generation of Aβ via the CREB/PGC-1α and SIRT1/PGC-1α signaling pathways [309]. Rhein promoted mitochondrial biogenesis through upregulating protein expression of SIRT1 and its downstream PGC-1α and NRF1, thereby attenuating Aβ_1-42_ oligomer-induced neuronal apoptosis [310, 311]. In addition, Dezfouli et al. observed increased protein expression of SIRT1 and TFAM, along with elevated mtDNA copy numbers, in the hippocampus of aβ-injected rats treated with melatonin for 14 consecutive days. However, these effects were interdicted by the administration of EX527, highlighting that SIRT1 signaling was implicated in the neuroprotective role of melatonin [312]. Sesamol, a nutritional component of sesame, ameliorated neuron damage and cognitive deficits in mice fed a high-fat and high-fructose diet, partly by enhancing the mRNA levels of SIRT1 and PGC-1α [313]. Honokiol, a natural product extracted from the bark of *Magnolia officinalis*, dramatically attenuated cognitive and synaptic impairments through enhancing the protein expression and activity of SIRT3 and activating mitophagy and mtUPR in male APP/PS1 mice [274]. Fingolimod rescued spatial memory impairment in a rat model of chronic cerebral hypoperfusion, which is associated with the regulatory role of SIRT3 in mitochondrial dysfunction and neuroinflammation [314]. Moreover, administration of nicotinamide mononucleotide, a NAD^+^ precursor, enhanced mitochondrial NAD^+^ pools in the hippocampus and triggered a global reduction in SIRT3-mediated mitochondrial protein acetylation. Therefore, the interaction between phosphorylated DRP1 and mitochondria was decreased, resulting in less mitochondrial fragmentation [315].

Additionally, SIRT1-mediated mitochondrial biogenesis has shown neuroprotective effects on dopaminergic neurons in PD models [316]. Urolithin A, a gut metabolite produced from foods containing ellagic acid, like walnuts, berries, and pomegranates, had a protective role in PD by markedly strengthening mitochondrial biogenesis via the SIRT1/PGC-1α pathway and ameliorating 6-OHDA-induced mitochondrial dysfunction [317]. Embelin is a natural product similar in structure to ubiquinone. Rao et al. found that embelin treatment promoted mitochondrial biogenesis through upregulating the protein levels of pAMPK, SIRT1, and PGC-1α [318]. β-Lapachone, a compound extracted from Lapacho bark, upregulated SIRT1 expression, CREB phosphorylation, and PGC-1α deacetylation, inhibiting HD phenotype [319]. Ghrelin, a brain-gut peptide, attenuated MPTP/MPP^+^-induced neurotoxicity by activating the AMPK/SIRT1/PGC-1α signaling pathway and promoting PINK1/Parkin mitochondrial translocation [252].

Furthermore, a small molecule inhibitor DDQ-treated Tau (P301L) transgenic mouse model showed higher levels of mitochondrial biogenesis and fusion, as well as higher mRNA and protein levels of sirtuins compared to untreated Tau mice [320]. Moreover, synthetic compounds, such as AGK-2 and AK-7 (SIRT2 inhibitors), have neuroprotective effects. AGK-2 reduced astrocyte activation and proinflammatory molecule production in an *in vitro* model of AD [321]. Another study indicated that AGK-2 and AK-7 suppressed SIRT2 and ameliorated cognitive performance in AD mouse models [322]. All these findings broaden our current understanding that improved mitochondrial quality control is essential to prevent neurodegeneration.

#### 6.2.2 Clinical trials

Most evidence supporting the neuroprotective effects of sirtuin-targeted drug interventions comes from cell or animal experiments. Human studies evaluating the long-term impacts are particularly few, even inconsistent. Several clinical trials have demonstrated the therapeutic efficacy of sirtuin modulators in neurodegenerative diseases. For example, Turner et al. examined the tolerability and safety of resveratrol in a 52-week, randomized, double-blind, placebo-controlled, multicenter phase 2 trial of 119 patients with mild to moderate AD. The findings provided Class II evidence that resveratrol is safe for AD patients and well tolerated [323]. Moreover, in a retrospective study of AD subjects (NCT01504854), resveratrol treatment markedly decreased the levels of metalloproteinase-9, fibroblast growth factor-2, macrophage-derived chemokines, and IL-4 at 52 weeks compared to placebo [324]. These compelling findings support the idea that sirtuins may be potential therapeutic targets for mitochondrial dysfunction and neurodegenerative disorders. However, future clinical trials with larger samples and longer durations are needed to explore the neuroprotective benefits of sirtuins for neurodegenerative diseases.

## 7. Conclusions and perspectives

Neurodegenerative diseases are characterized by a slow, progressive loss of selective neuronal cell populations and neuronal dysfunction. The prevalence and incidence of neurodegenerative disorders increase significantly with age. However, there are still not fully effective and definitive pharmacological treatments for neuro-degenerative diseases. There is strong evidence that mitochondrial dysfunction occurs early on and acts causally in the pathogenesis of all major neuro-degenerative diseases. Dysregulated sirtuin activity is strongly related to mitochondrial dysfunction, which provides an attractive direction for the intervention and treatment of neurodegenerative disorders. Sirtuins are promising therapeutic targets for neurodegenerative diseases. They are highly conserved NAD^+^-dependent enzymes that regulate longevity and multiple physiological processes, such as mitochondrial biogenesis, autophagy, mitochondrial dynamics, and mtUPR, through catalyzing a wide range of specific substrates. Given the interesting and encouraging results for regulating sirtuin activity through exercise training, calorie restriction, and the development of sirtuin modulators in cellular and animal models of neurodegenerative diseases, as well as the results of clinical trials, sirtuins emerge as potential therapeutic targets for mitochondrial quality control and neurodegenerative disorders. Compelling evidence for the protective effects on neurodegeneration, combined with continued research on the structure, transcriptional regulation, post-transcriptional regulation, and post-translational modifications of sirtuins, and further exploration of the detailed mechanisms of action of sirtuin-mediated mitochondrial quality control in neurodegenerative diseases, make the targeting of sirtuins a promising strategy for the treatment of neurodegenerative disorders. Future work is needed to elucidate the exact molecular mechanisms among sirtuins, mitochondrial function, and neurodegenerative diseases. Additionally, exploration of novel sirtuin modulators and the development of comparative studies of cells, animals, and clinical trials may facilitate the discovery and development of effective anti-neurodegeneration drugs.
